# Primary and Secondary Emissions of VOCs and PAHs in Indoor Air from a Waterproof Coal-Tar Membrane: Diagnosis and Remediation

**DOI:** 10.3390/ijerph182312855

**Published:** 2021-12-06

**Authors:** Rafael Piñeiro, Eva Jimenez-Relinque, Roman Nevshupa, Marta Castellote

**Affiliations:** 1 Institute of Construction Science “Eduardo Torroja”, IETcc (CSIC), 28033 Madrid, Spain; rpineiro@ietcc.csic.es (R.P.); eva.jimenez@csic.es (E.J.-R.); r.nevshupa@csic.es (R.N.); 2 Arquitecture School, Madrid Polytechnic University, 28040 Madrid, Spain

**Keywords:** VOCs, PAHs, emissions, indoor, coal-tar membrane, terrace, diagnosis, remediation

## Abstract

Primary and secondary emissions of volatile organic compounds (VOCs) and polycyclic aromatic hydrocarbons (PAHs) from a waterproof coal tar membrane and their effect on the indoor air quality were investigated through a case study in a residential building situated in Madrid, Spain. The air contaminants were analyzed in situ using photoionization method and several samples of contaminants were taken using three sorbents: activated carbon, XAD2 and Tenax GR. It was found that various VOCs such as toluene, p- and m-Xylene, PAHs such as naphthalene, methyl-naphthalenes, acenaphthene, acenaphthylene, phenanthrene and fluorine, volatile organic halogens including chloroform and trichlorofluoromethane, and alkylbenzene (1,2,4-trimethylbenzene) were found at concentrations, which exceeded the limits established by international and national agencies (WHO, EPA, OSHA). Some of the above organic compounds were found also in the samples of construction and building materials, which were obtained at different heights and places. The analysis of possible sources of the contaminants pointed at the original coal-tar membrane, which was applied on the terrace to be waterproof. During a posterior reparation the membrane was coated with a new one that hindered dissipation of emitted contaminants. The contaminants leached out and were absorbed by construction materials down in the dwelling. These materials then acted as secondary emission sources. To remediate the emission problem as the contaminated materials were removed and then a ventilation system was installed to force the gasses being emitted from the rest of contaminated slab outside. Follow-up has validated the success of the remediation procedure.

## 1. Introduction

Polycyclic aromatic hydrocarbons (PAHs) are a group of organic compounds with two or more fused aromatic rings. Among the PAHs, the Environmental Protection Agency (EPA) designated 16 high priority pollutants including naphthalene, acenaphthylene, acenaphthene, fluorene, phenanthrene, anthracene, fluoranthene, pyrene, benzo[a]anthracene, chrysene, benzo[b]fluoranthene, benzo[k]fluoranthene, benzo[a]pyrene, benzo[g,h,i]perylene, indeno [1,2,3-c,d]pyrene, and dibenzo[a,h]anthracene. These 16 PAHs are of special environmental concern because of their potential toxicity for humans, flora and fauna, and their prevalence and persistence in the environment. The PAHs can be found in air, soil, water, plants and even in food [1]. Furthermore, some of the PAHs are suspicious or proved carcinogens [2]. Therefore, during the last decades, there has been a growing concern about the amount of the PAHs in both indoor and outdoor air [3].

During the last decades, much more efforts were made to improve the indoor air quality [4,5,6] that involves the development of low emitting construction materials [7,8,9,10,11], rational ventilation [12] and the understanding of the chemistry of the indoor environment [13,14,15,16] including that of the PAHs [17]. Recent systematic literature reviews [18,19] have analyzed the PAHs sources and their effects on human health. The two main PAHs sources, which are related to traffic emission and coal combustion, were identified in case studies carried out in schools in Serbia, in buildings in China [20,21]. Zorn et al. [22] proposed assessing PAHs in indoor air. Detailed examination of naphthalene by Batterman et al. [23] was carried out both outdoor and indoor in 288 houses in Michigan. The median outdoor concentration was 0.15 µg/m^3^, while indoor was 0.89 µg/m^3^, being highly skewed with levels that reached 200 µg/m^3^ with the consequent increase in cancer risk of the users of those buildings. Furthermore, the house dust [24] was recognized as an important pathway for human exposure to contaminants and PAHs [25]. The recent study [26] showed that, in residences situating near coal-tar-sealed pavement, house dust might be the primary and biologically relevant way for humans exposure to PAHs, especially in young children. The works [27,28] concluded that the concentration of PAHs in settled house dust significantly depended on the presence of coal-tar on the parking lot. In turn, the intensity of urban land use near the residence had a weak influence on the PAHs concentrations, while other variables such as carpeting, frequency of vacuuming, and indoor burning, did not correlate with the PAHs concentrations. The PAHs concentration in house dust were 25 times higher in the buildings situating close to parking lots sealed with coal-tar than in twin unsealed residents [27].

The coal tar-based products are largely used as protective coatings for concrete structures and in waterproof systems in basements, roofs and terraces [29]. Normally, the coal-tar-based sealants contain more than >50,000 mg/kg of the 16 PAHs [26,30] that makes them potentially a source of toxic emission. The meta-analysis performed in [31] provided evidences supporting the hypothesis that the exposure to the combination of various coal tar-related PAHs can explain most if not all cases of lung cancer among workers of asphalt roofing and paving. Van Metre et al. [32] carried out a quantitative study of PAH volatilization after application of coal-tar-based pavement. The striking result was that the volume of annual PAH emission related to the application of coal-tar-based pavements in the Unites States exceeded the volume of PAHs emission by the exhaust of road transport. The adverse effects to the human health of PAHs, their cyto- and genotoxicity were investigated in various studies [33,34,35]. The PAHs released from coal tar-based coatings persist in seawater for 6–96 h and can be accumulated in marine organisms such as oysters [36].

While the risks related to the use of coal-tar products are supported by solid proofs, very scarce studies of contamination in buildings, which can be associated with the emission from coal-tar waterproofing membranes installed in terraces [37]. This work describes a case study of acute contamination in a residential building, which made it temporarily inhabitable. The base analysis, the diagnosis, the proposed solution and the follow up tests are described.

## 2. Materials and Methods

### 2.1. Description of the Problem

**Figure 1:**
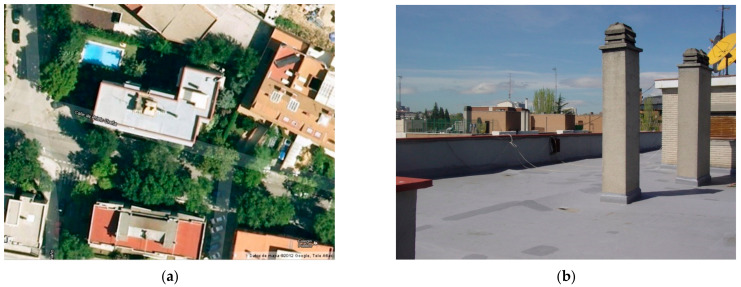
(**a**) Aerial photograph of the building in Madrid; (**b**) detail of the building roof.

The flat addressed in this study is situated in Madrid, on the top floor of an L-shaped building constructed in 1973 (Figure 1). The flat facing southeast is comprised of a living room, three bedrooms, a kitchen, a bathroom, a toilet and two balconies. During the initial interview with the owner, it was found that the flat had become uninhabitable due to the severity of the environment-induced health issues, including headaches, eye, nose and throat irritations and shortness of breath. The relevant data were collected during the site-visits in order to determine the constructional composition of the building and to make a correct diagnosis of the problem. It was observed that the usable roof on the top of the flat was waterproofed in the course of a freehold maintenance operation. The core samples were extracted to determine the composition of various layers of the roofing materials. The original structure consisted of a cast-in-place, thin-brick pan form, a light-concrete deck, a layer of lightweight mortar, a layer of levelling mortar to create a slope and a coal-tar-based waterproof membrane.

**Figure 2:**
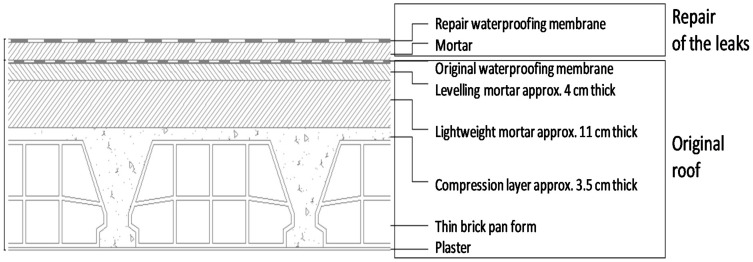
Cross-sectional schematic drawing of the roof.

A new weatherproof membrane was placed on the top of the existing material in subsequent repairs, as illustrated in Figure 2. That operation entailed placing levelling mortar on the top of the original waterproof membrane, which was further coated with a new weatherproof membrane.

### 2.2. Environmental Assessment

1. The air-borne compounds susceptible to photoionization were assessed in all the rooms using a Photovac 2020 hand-held photoionization detector (PID). The efficiency of detection of the PID depends on the ionization potential, IP (eV), of the target compound. The equipment employed a 10.6 eV UV lamp. Therefore, any compounds with an IP smaller, than 10.6, could be detected. According to [38], this includes most PAHs. It should be stressed that the measurement results have to be taken with caution, mainly as indicative values, since the photoionization response of various compounds significantly differ.
2. The samples of air-borne contaminants were collected separately for the living room, bedroom D3 and the inter-joist pan forms in the structural ceiling. From the ceiling, the air samples were obtained at different heights 1.5 m from the floor. The flat was not ventilated for 12 h prior to the sampling. The air was pumped through three different sorbents using a pump Buck-basic supplied by A.P. Buck Inc., Orlando, FL, USA. Three types of sorbents were used: activated carbon supplied by Drager (AC), XAD2 and Tenax GR supplied by Supelco. The sorbents were chosen in order to capture the largest possible number of various VOCs and PAHs. The analysis of the adsorbed contaminants was made using gas chromatography coupled to a mass spectrometer (GC-MS) by ALcontrol Laboratories, in Rotterdam, Netherlands. The measurements were conducted in three campaigns in April, June and September 2012, respectively.

### 2.3. Pollutant Assessment in Construction Materials

**Figure 3:**
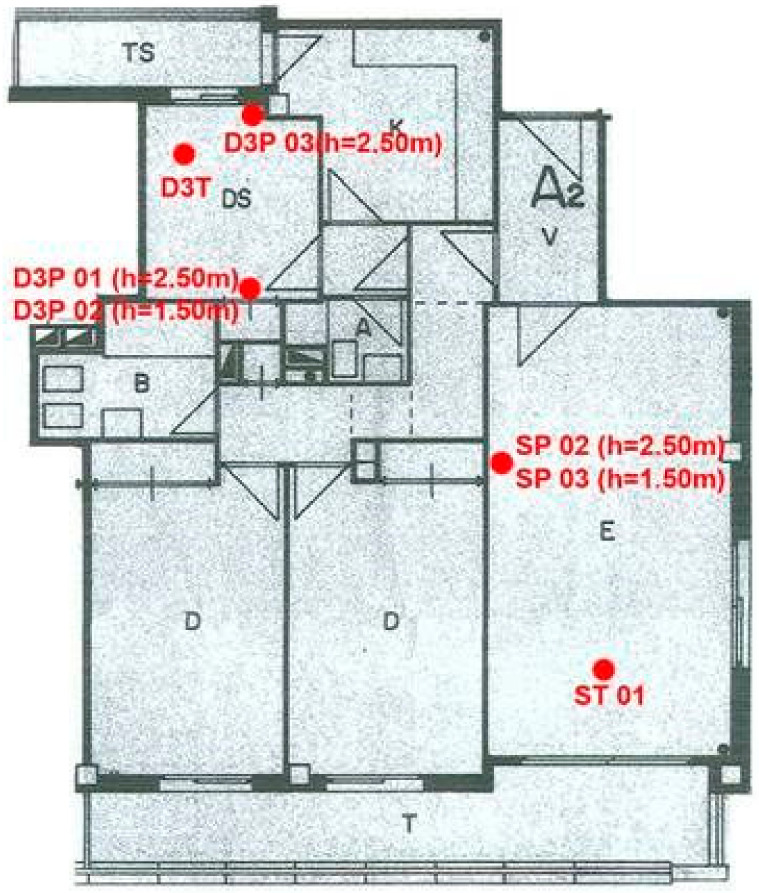
The positions of the core sampling points.

The samples of the building materials or cores were collected from the ceilings and walls in various rooms of the flat and then analyzed to determine the presence of volatile (VOC) or semi-volatile (SVOC) organic compounds. No samples were taken of the timber floors, window frames, and doors. The positions of the core sampling points are shown in Figure 3. More specifically, the cores were sampled in the living room and the smallest bedroom, which was denoted D3, using a circular coring bit driven by an electrically powered percussion drill. No duplicate samples were considered because in a real contaminated site assuming that two samples are identical might not be correct. In the living room, one core was obtained on the ceiling (core ST1) and the other two were extracted from the wall, at the same vertical line, but on different heights: SP02 at 2.5 m from the floor and SP03 at 1.5 m from the floor. In D3 bedroom four core samples were obtained at the following locations: D3T was extracted from the ceiling, D3P01 and D3P02 were extracted from the wall at the same heights as SP02 and SP03, correspondingly, and the fourth sample, D3P03, was extracted from the internal part of the outwall, next to the exit to the balcony, at the height of 2.50 m and to the depth of the air chamber.

The air coming out from the openings, which left after extracting the cores, was also analyzed using the methods of absorbent tubes and photoionization described above.

## 3. Results

### 3.1. Contaminants in the Air

#### 3.1.1. Photoionisation Analysis

The mean and standard deviation of the total isobutylene-equivalent concentration of the air contaminants, which were obtained in the three campaigns, are summarized in Table 1. The air temperature and relative humidity are also given. The results obtained for the air coming out from the drilling holes are listed in Table 2.

The increase of the total contaminants concentration during the second campaign can be associated with the seasonal increase of both the air temperature and the daylight hours that could enhance the emission rate of volatiles from the building materials in June in comparison with the other two campaigns. For example, the saturated vapour pressure of naphthalene increases 2.7 times as the temperature increases from 20 °C to 30 °C [39]. This is close to 2.3 increase in total PAHs concentration that was determined between the first and the second campaigns.

#### 3.1.2. Analysis of the Contaminants Captured by the Sorbents

Table 3 shows the compounds, which were identified among the contaminants captured by various sorbents in the indoor air, while Table 4 shows the data for the air extracted from the inter-joist pan form. The main components were naphthalene, volatile hydrocarbons and PAHs. Smaller amounts of toluene, p- and m-Xylene, methyl-naphthalenes, acenaphthene, acenaphthylene, phenanthrene, fluorene, chloroform, trichlorofluoromethane and 1,2,4-trimethylbenzene were also detected. For the air extracted from the ceiling inter-joist hollow brick the composition was quite similar except the absence of volatile organic halogens and alkylbenzenes and the presence of fluoranthene, pyrene, 1,4-dichlorobenzene and 1,3,5-trimethylbenzene. Several compounds absorbed on Tenax GR sorbent such as biphenyl, diethyl phthalate, dibenzofuran among others could not be quantified with sufficient accuracy.

The partial pressure of naphthalene can be estimated from its mass concentration, *C*, in air using the following expression:

(1) $$p_{naph}=\frac{C}{M}RT$$

where *M* is the molar mass of naphthalene (*M* = 128 g/mol), *R* is the universal gas constant (*R* = 8.31 J/(mol K)) and *T* is the temperature.

For *C* = 154 mg/m^3^ the partial pressure was 3 Pa that means that naphthalene vapour was close to saturation at 20 °C.

### 3.2. Building Materials

Table 5 summarizes the results of determination of contaminants concentrations in the core samples extracted at different locations in the flat. The results are classified according to the types of the construction materials. Additionally, the concentrations of the contaminants in the air, which were measured using PID method just after removal of the corresponding core sample, are also presented. The highest concentrations of the contaminants were found in the layer containing the initial coal-tar waterproof membrane. The PAHs were dominating among all other detected contaminants.

## 4. Discussion

### 4.1. Diagnosis

During the first inspection, the characteristic smell of naphthalene could be detected both in the lift and on the stair around the fourth floor. On the stair the smell was more intensive and could be noticed even one floor down. Inside the flat, which to the date of the first inspection had not been ventilated for unidentified number of days, the air quality was so bad that it was not possible to continue the inspection without the use of the personal protection equipment including anti-organic compound face masks and goggles. Further analysis confirmed the presence of volatile and semi-volatile organic contaminants in both the indoor air and construction materials. The primary source of pollution could be undoubtedly traced to the original waterproof membrane on the roof. The predominant pollution components, namely polycyclic aromatic hydrocarbons and especially naphtalene, were associated with the coal tar used for fabrication of the original waterproof membrane. Since during a posterior rehabilitation the original waterproof membrane was coated with a layer of levelling mortar and a new waterproof membrane, the latter must have formed a vapor barrier that hindered the emission of the volatiles to the outdoor environment and their dissipation. Therefore, the contaminants could percolate through the construction materials into the floor situated beneath the roof.

**Figure 4:**
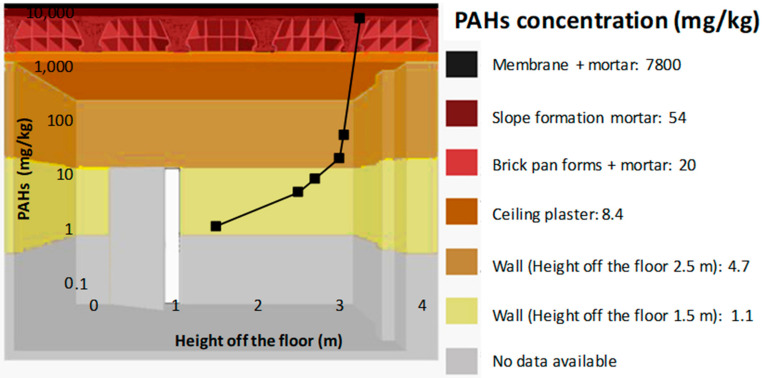
Variation in PAH concentration in materials with height.

Figure 4 shows the distribution of the total concentration of the 16 PHAs with the height above the floor. Although these data should be taken with caution because of the small volume of the measured values, there is no doubt that there must be only one primary emission source and that it situated on the roof coinciding with the original waterproof membrane.

From the comparison of the PAHs concentrations measured at various heights it was suggested that percolation of the contaminants into mortars, concretes, brick pan forms, plaster finishes and other materials employed in the ceiling and walls could be assisted by water, which leaked from the roof into the flat during several episodes in the past. On the other hand, the presence of notable amounts of the PAHs, especially naphthalene, acenaphthene and phenanthrene, in the walls at the lower measured level from the floor, 1.5 m, may also point at the possible readsorption of the emitted components on previously uncontaminated surfaces. It cannot be discarded that high emission rates of the PAHs from the primary and secondary sources together with poor ventilation might lead to massive migration of the contaminants all over the entire flat that was indirectly confirmed by the persistent smell and by the fact that the partial pressure of naphthalene vapours was close to saturation. The amount of PAHs readsorbed on various surfaces, *q*, can be assessed using Langmuir’s adsorption isotherm:

(2) $$\frac{q}{q_{m}}=\frac{bC}{1+bC}$$

where *q_m_* is the maximal adsorption capacity of the surface and *b* is the constant.

Puszkarewicz et al. [40] studied adsorption of naphthalene on activated carbon and minerals (clinoptilolite) and reported the following values of the parameters: for carbons *b* = 0.17–0.20 dm^3^/mg, *q_m_* = 19–32 µg/m^2^ and for the mineral *b* = 0.102 dm^3^/mg, *q_m_* = 2.78 µg/m^2^. Then, assuming *C* = 154 mg/m^3^ the concentration of adsorbed naphthalene on a carbon surface would be 0.51–0.86 µg/m^2^ or 2.7% of its adsorption capacity, while for the mineral *q* = 0.043 µg/m^2^ or 1.5% of its adsorption capacity. Considering a specific surface area of constructional materials about 100 m^2^/g [41] the concentration of absorbed naphthalene could be as high as 4.3–86 mg/kg. These values agree the results of experimental measurements of PAHs in the samples extracted from the walls of the flat. Furthermore, the sojourn time of adsorbed naphthalene molecules for the typical range of activation energy for desorption 99.4–190 kJ/mol [42] is too large (10^5^–10^15^ s) to expect that its concentration can decrease by evaporation below the exposure limit in reasonable time.

**Figure 5:**
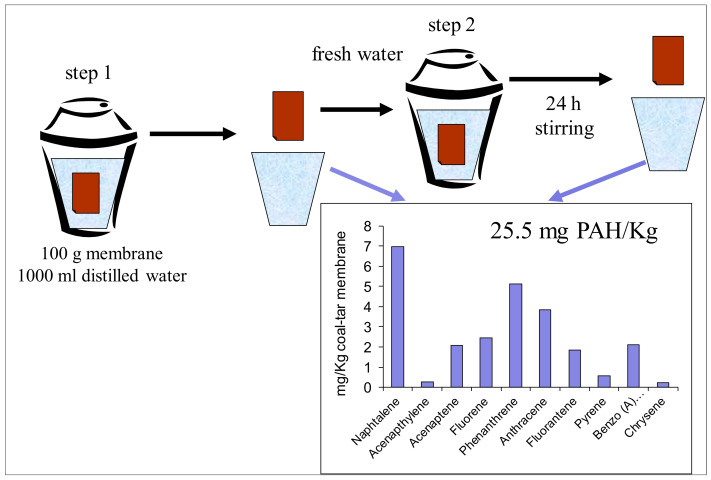
Schematic drawing of the experimental simulation of leaching of the contaminants from a coal-tar membrane in water and the obtained results.

In order to prove the hypothesis on the effect of water leaks in spreading the contaminants over the construction materials of the flat, the experimental simulation schematically shown in Figure 5 was carried out. A piece of a similar aged coal-tar membrane was placed into distilled water for two periods. The solid-to-liquid ratio was 1:10. In both cases, the analysis showed the presence of a significant amount of PAHs in water after the tests yielding the total concentration 25.5 mg/Kg. The relative concentrations of various components also agreed the composition of the contaminants in the samples of construction materials obtained in the flat.

A number of existing guides and quality standards describe the criteria that can be used to appraise the air quality. The standards referring to outdoor air quality aim to protect the population from adverse effects on health or discomfort as a result of exposure to environmental pollution. Such standards are often applied as a reference to define indoor air quality. For compounds not listed in the aforementioned guides, the recommendation is to apply the occupational exposure limit values corrected by a factor of 1/10 [43].

The legislation on the ceiling concentrations in place for this type of analysis includes different Indoor air quality guides and standards, which in some cases recommend different threshold limits [44]. In this study, the following guides were used: the National Occupational Health and Safety Institute’s (INSHT) occupational exposure limit values [45], the National Institute for Occupational Safety and Health’s (NIOSH) and the Occupational Safety and Health Administration’s indoor environmental quality reference values) [46,47], Environmental Protection Agency (EPA) and World Health Organisation (WHO) guidelines [48] and the air quality values set out in a report issued by the City of Madrid’s Department of the Environment [49]. As a rule, the limit values established by the workplace legislation are higher, than those listed in indoor environmental quality guides, inasmuch as workers are not supposed to be as sensitive as children or the elderly.

The naphthalene concentration in both the living room and bedroom notably exceeded the World Health Organization’s and the OSHA’s indoor environmental limits. Both 1,2,4-trimethylbenzene and 1,3,5-trimethylbenzene exceeded the 0.7 µg/m^3^ ceiling allowed by the Environmental Protection Agency (EPA). A number of volatile organic compounds in the air trapped in the ceiling slab pan forms were observed to exceed the general limits. The value recorded for toluene in the living room exceeded the 5 µg/m^3^ ceiling for outdoor air defined by the City of Madrid’s Department of the Environment. Phenanthrene doubled the OSHA 200 µg/m^3^ limit, whilst acenaphthene nearly was three-fold higher than the recommended value by OSHA. Fluorene was twice as high as the limit 100 µg/m^3^ established by the National Institute for Occupational Safety and Health’s (NIOSH). The volatile hydrocarbon (C6-C12) concentration was found to be very significant and much above the limit values specified for outdoor air in the City of Madrid.

Taken together the above results led us to the conclusion that the contamination of the flat was unacceptably high and it could have severe impact on the health of its residents. Such situation should not be overlooked, and the remediation measures to improve the situation were developed and implemented.

### 4.2. Remediation

**Figure 6:**
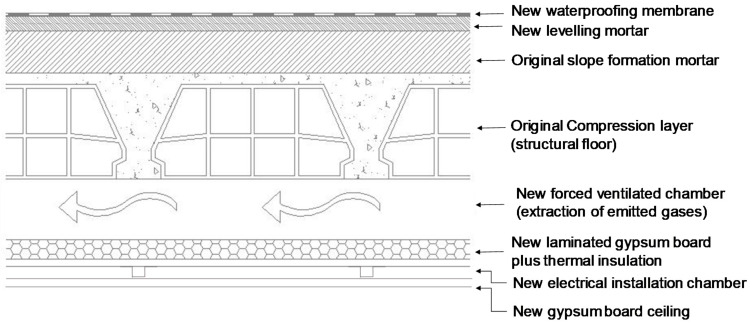
The constructive solution for remediation implying partial demolition of the roof.

The original waterproof membrane installed on the terrace of the flat was identified as the primary source of the PAHs emission. The building materials in various parts of the flat, which were contaminated due to water leakage or readsorption of the contaminants from the air, were considered the secondary emission sources. To eliminate or significantly reduce the emissions from these sources, both the replacement of the original waterproof membrane on the roof and the interior rehabilitation were necessary. During the remediation stage, the waterproof membrane was completely removed from the roof except for the booth of the lift machinery and the stairwell. The latter two elements being the common elements of the building were excluded from the activity since the permission from owners of the building was not obtained. In turn, the owners of the flat decided to rehabilitate and reform the flat at the same time including elimination of all interior distribution elements and their coverings and replacement with the new ones. Considering that, despite high degree of contamination, the compression layer being a part of important structural element could not be removed in a simple rehabilitation project, an alternative wave to reduce the primary source of the contaminant emission was chosen. It involved the creation of a false ceiling consisting of a watertight chamber to avoid possible future water leaks from the roof into the flat and forced ventilation (Figure 6). By doing so the emitted contaminants could be extracted and dissipated in the outdoor environment that significantly reduced their penetration into the flat.

After one year of the completion of the renovation work, a new inspection was carried out, in September 2015, to evaluate the effectiveness of the measures adopted. For this purpose, the samples of the air-borne contaminants were collected at three different locations: the living room, the exhaust from the ventilated chamber of the false ceiling and the roof of the staircase, where the original membrane was not removed, using the sorbent tubes. The compounds detected in the analysis of the samples are given in Table 6.

The concentration of air-borne volatile and semi-volatile compounds in the flat (living room) significantly decreased. For all compounds the concentrations were far below the exposure limits established by the national and international Indoor Environment Quality regulations and recommendations. Furthermore, for many compounds the concentrations were below the detection limits. Toluene had slightly higher concentration (0.041 µg/m^3^) in the living room, than in other two locations, that can be associated with residual contamination of some of the flat elements. The contaminants concentration on the landing of the roof floor also significantly decreased in comparison with the initial study. At this place, only toluene and naphthalene were identified. The concentration of the former was much below the exposure limits, but the concentration of naphthalene did not significantly change since the initial study and exceeded the OSHR and WHO limits. It was concluded that the roof of the staircase cassette, which conserved the original waterproof membrane, continued emitting volatile and semi-volatile organic compounds into the environment of the dwelling. Likewise, high contaminants concentration was observed at the air exhaust from the chamber in the false ceiling, most of them exceeding the corresponding exposure limits. These findings demonstrated that the adopted solution was effective remediate the emission of the contaminants even without the complete elimination of the primary source.

**Figure 7:**
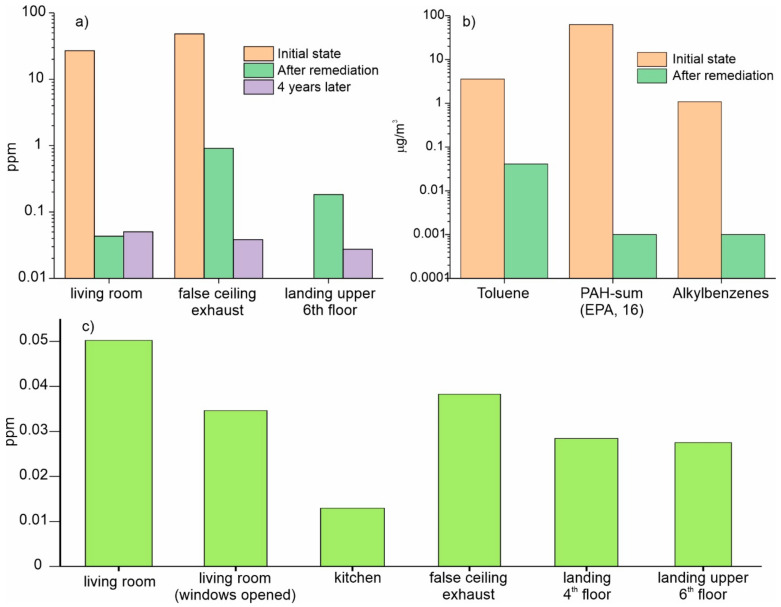
Graphical comparison of the results in the three inspections (**a**) PID readings (isobutylene equivalent concentration), (**b**) the amount of different type of air-borne compounds in the living room (µg/m^3^), (**c**) PID readings 4 years after the remediation tests at different locations.

In July 2019, 4 years after the completion of the remediation works, a final inspection was carried out. The total amount of the compounds, which could be photoionized, was measured at various points of the dwelling yielding values below 0.06 ppm (isobutylene equivalent). Figure 7a–c summarize the obtained results.

## 5. Conclusions

The following conclusions were drawn:

- The primary source of contamination was identified as the PAHs (polycyclic aromatic hydrocarbons) emitted by the original rooftop coal tar-based waterproof membrane. The contamination was attributed to the repair of the original roof, over which a layer of mortar and a liquid waterproof membrane were placed as a solution to the water leakage problem. The second membrane prevented the contaminants to be dissipated in the outdoor environment, and they migrated into the interior of the house, which process was accelerated by water leaks. The contaminated building materials were the secondary emission source of air-borne contaminants.
- Indoor naphthalene concentrations exceeded the limits laid down by both the WHO and OSHA. The levels of 1,2,4-trimethylbenzene in the air exceeded the EPA’s limit value. The air in the ceiling slab pan forms contained volatile organic compounds such as toluene, acenaphthene, phenanthrene, fluorene, 1,3,5-trimethylbenzene and volatile hydrocarbons (C6-C12) above the established limits.
- The remediation of the contamination problem implied removing the primary and secondary emission sources to the highest possible extent, and creation of the alternative emission ways using an additional ventilated chamber at a false ceiling with forced extraction of the air. The follow up tests validated the remediation solution.

## Figures and Tables

**Table 1:** Photoionization readings in the indoor air.

Campaign	Concentration ^(1)^ (ppm): Mean ± sd		*T* (°C)	RH (%)
	Living Room (E)	Bedroom (D)		
1	11.7 ± 1.57	10.7 ± 1.31	18.0 ± 3.97	24.8 ± 11.1
2	27.0 ± 2.75	20.5 ± 3.74	27.8 ± 2.4	24.4 ± 7.28
3 ^(2)^	9.05 ± 4.87	9.25 ± 3.32	24.5 ± 2.89	44.4 ± 2.47

^(1)^ Isobutylene equivalent. ^(^^2)^ The readings obtained after extracting the cores.

**Table 2:** The total concentration of contaminants in the air coming out from the boreholes measured using photoionization method.

Borehole	Campaign	Concentrations ^(1)^ (ppm)		*T* (°C)	RH (%)
Location		Living Room (E)	Bedroom (D)		
Ceiling	2	48.1	32.8	32.8	34.1
Wall, H = 2.5 m		14.8	19.2		
Wall, H = 1.5 m		22.8	17.2		
Ceiling	3	26.3	23.8	24.8	40.6
Wall		9.25		24.5	44.4

^(1)^ Isobutylene equivalent.

**Table 3:** VOC and SVOC concentrations (µg/m^3^) in the air.

	Living Room		Bedroom	
Type of Sorbent	AC	XAD2	XAD2	Tenax GR
Volatile aromatic compounds				
Toluene	3.60			0.210
p- and m-Xylene	1.92			
Naphthalene	154			2.26
Polycyclic aromatic hydrocarbons				
Naphthalene		48.0	16.3	2.26
Acenaphthene		13.2	13.3	0.088
Phenanthrene		1.32	1.14	
Acenaphthylene		0.18	0.11	0.065
Fluorene		0.16	0.12	
Fluoranthene				
Methylnaphthalenes				1.47
PAH-total (VROM. 10)		49.3	17.3	
PAH-total (EPA. 16)		62.5	30.3	
Volatile organic halogens				
Chloroform	1.26			
Trichlorofluoromethane	0.94			
Alkylbenzenes				
1.2.4-Trimethylbenzene	1.08			
Hydrocarbons				
Volatile hydrocarbons (C6-C12)	168			

**Table 4:** VOC and SVOC concentrations (µg/m^3^) in the air extracted from the inter-joist pan form.

	Living Room			Bedroom	
Type of Tube	AC	XAD2	Tenax GR	XAD2	Tenax GR
Volatile aromatic compounds					
Toluene	5.29				
p- and m-Xylene	2.58				
Naphthalene	6152		8.05		3.69
Polycyclic aromatic hydrocarbons					
Naphthalene		21.2	8.05	0.513	3.69
Acenaphthene		511			0.112
Phenanthrene		401			
Fluorene		175			
Fluoranthene			7.44		4.47
Pyrene		6.49			
PAH-total (VROM. 10)		496		0.513	
PAH-total (EPA, 16)		1167		0.513	
Chlorobenzenes					
1,4-Dichlorobenzene	0.62				
Alkylbenzenes					
1,3,5-trimethylbenzene	1.17				
Hydrocarbons					
Volatile hydrocarbons (C6-C12)	3076				

**Table 5:** VOC and SVOC concentrations in the construction materials.

Location	Living Room-Ceiling				Living Room-Wall		Bedroom-Wall	
Material	Plaster	Leveling Mortar	Pan Forms +Mortar	Membrane +Mortar	Plaster +Brick	Plaster +Brick	Brick	Paint +Plaster +Brick
Sample	ST01-01	ST01-04	ST01C-06	ST01C-08	SP02-01	SP03-02	D3P02-02	D3P03-01
Dry matter wt%	88	94.5	98.8	96.4	100	100	92	85.5
PID values (ppm)	24.55	16.56			11	32.6	17.1	
	Polycyclic aromatic hydrocarbons (mg/kg dry matter)							
Naphthalene	0.76	2.4	0.99	230	0.62	0.13	0.16	0.35
Acenaphthylene	0.09	0.05	0.82	6.6	<0.03	<0.03	<0.02	<0.02
Acenaphthene	2.2	7.8	2.8	540	1.8	0.72	0.35	0.88
Fluorene	0.25	1.2	0.35	350	0.11	<0.03	0.03	0.02
Phenanthrene	4.9	28	13	2300	2	0.19	0.18	0.27
Anthracene	<0.02	1.4	0.3	460	<0.03	<0.03	0.02	<0.02
Fluoranthene	0.09	8	1.1	1300	0.08	0.04	0.09	0.03
Pyrene	0.03	4	0.34	780	0.05	<0.03	0.06	<0.02
Benzo(a)anthracene	<0.02	0.7	<0.02	340	0.03	<0.03	0.02	<0.02
Chrysene	<0.02	0.58	<0.02	290	0.03	<0.03	0.02	<0.02
Benzo(b)fluoranthene	<0.02	0.31	<0.02	360	0.03	<0.03	0.02	<0.02
Benzo(k)fluoranthene	<0.02	0.14	<0.02	160	<0.03	<0.03	<0.02	<0.02
Benzo(a)pyrene	<0.02	0.04	<0.02	260	<0.03	<0.03	<0.02	<0.02
Dibenzo(a,h)anthracene	<0.02	<0.02	<0.02	49	<0.03	<0.03	<0.02	<0.02
Benzo(g,h,i)perylene	<0.02	0.04	<0.02	170	<0.03	<0.03	<0.02	<0.02
Indene(1,2,3-cd)pyrene	<0.02	0.05	<0.02	190	<0.03	<0.03	<0.02	<0.02
PAH-total (VROM, 10)	5.8	41	16	5700	2.7	0.36	0.52	0.64
PAH-total (EPA, 16)	8.4	54	20	7800	4.7	1.1	0.98	1.5

**Table 6:** The concentrations of the air-borne contaminants one year after the remediation works.

Concentrations (µg/m^3^)			
	Living Room	False Ceiling Exhaust	Staircase Landing on the Upper (6th) Floor
Toluene	0.041	0.003	0.003
Naphthalene	0.001	134.36	49.5
1,3,4-Trimethylbenzene	0.001	0.003	
Acenaphthene		71	
Phenanthrene		52	
Fluorene		24	
